# Neocnidilide and 6-Gingerol as Key Bioactives in Fresh and Dried *Centipeda minima*: Distinct Th1/Th2 Modulation via NF-κB/JAK-STAT Pathways for Allergic Rhinitis Therapy

**DOI:** 10.3390/ijms26178678

**Published:** 2025-09-05

**Authors:** Yamin Zhang, Jiajia Lin, Xiaomei Xu, Xuehua Lu, Lisha Li, Yuezhen Yang, Wenjin Lin

**Affiliations:** 1 Fujian Key Laboratory of Medical Analysis, Fujian Academy of Medical Sciences, Fuzhou 350001, China; xuxiaomei@fjms.ac.cn (X.X.); lxh6675@fjms.ac.cn (X.L.); lisa_faoms@fjms.ac.cn (L.L.); 2 School of Pharmacy, Fujian Medical University, Fuzhou 350122, China; ljj55515@163.com (J.L.); louise2112@163.com (Y.Y.); 3 College of Bee Science and Biomedicine, Fujian Agriculture and Forestry University, Fuzhou 350002, China

**Keywords:** *Centipeda minima*, fresh and dried products, allergic rhinitis, transcriptomics, widely targeted metabolomics

## Abstract

This study aimed to compare the therapeutic effects of fresh (CMF) and dried (CMD) *Centipeda minima* against allergic rhinitis (AR), elucidate their underlying molecular mechanisms, and identify the bioactive compounds responsible for their immunomodulatory actions. An ovalbumin-induced AR mouse model was treated with CMF or CMD extracts, followed by evaluation of nasal symptoms, serum biomarkers (IgE, histamine, cytokines), and nasal mucosa histopathology. Transcriptomics and widely targeted metabolomics were integrated with network pharmacology to identify differentially expressed genes and bioactive components, which were further validated in RAW264.7 and RBL-2H3 cells. CMF and CMD exhibited distinct anti-AR mechanisms: CMF predominantly suppressed Th2 responses (reducing IgE, IL-6, and histamine while elevating IL-10), whereas CMD enhanced Th1 activity (increasing IFN-γ). Metabolomic analysis revealed CMF was rich in amino acids while CMD contained higher flavonoids, with neocnidilide and 6-gingerol identified as key bioactive compounds that modulated TNF-α, IL-6, and IL-10 via NF-κB and JAK-STAT pathways. These findings demonstrate that CMF and CMD exert complementary anti-inflammatory effects through Th2 inhibition and Th1 activation, respectively, providing a molecular basis for the traditional use of *Centipeda minima* and highlighting its bioactive compounds as potential therapeutics for inflammatory diseases.

## 1. Introduction

Allergic rhinitis (AR) is an immunoglobulin E (IgE)-mediated inflammatory disease of the nasal mucosa triggered by exposure to allergens such as pollen, dust mites, and animal dander [1,2]. It affects approximately 10–40% of the global population, with particularly high prevalence among children, and poses a substantial socioeconomic burdens due to its associations with asthma, sinusitis, and sleep disorders [3,4,5]. The pathogenesis of AR is driven by a helper 2 (Th2)-skewed immune response, marked by elevated secretion of interleukin-4 (IL-4), IL-5, and IL-13, coupled with eosinophil infiltration and remodeling of the nasal mucosal [6,7]. Current mainstay treatments—including antihistamines and intranasal corticosteroids—often deliver only symptomatic relief and may lead to adverse effects upon long-term use [1,8,9]. These limitations have accelerated the search for safer and more effective alternatives, particularly bioactive compounds derived from medicinal plants, which demonstrate promising immunomodulatory properties and favorable safety profiles [10,11,12].

*Centipeda minima* (L.) A. Braun & Asch. (*Asteraceae*), known as “É Bù Shí Cǎo” (*Spreading sneezeweed*) in Chinese, is a traditional herbal medicine long used for relieving nasal obstruction, sinusitis, and allergic symptoms [13]. Historical records, including the “Four Tones Materia Medica” from the Tang Dynasty, note its ability to “unblock nasal passages, facilitate the opening of the nine orifices, and expel wind-phlegm”. The Compendium of Materia Medica (Ben Cao Gang Mu) from the Ming Dynasty further documented its efficacy in topical applications for nasal polyp elimination. Modern pharmacological studies have revealed that *Centipeda minima* exhibits anti-inflammatory, antioxidant and anticancer effects, largely attributed to its diverse phytochemical composition encompassing sesquiterpenes, flavonoids, and phenolic acids [14,15,16,17,18]. However, although previous research has focused primarily on volatile extracts of this herb [19,20], the pharmacological differences between its fresh (CMF) and dried (CMD) forms remain poorly understood. This represents a significant knowledge gap, given that post-harvest processing is known to substantially alter the composition and bioactivity of herbal products [21,22,23].

Recent advances in ethnopharmacology have underscored the potential of natural products in rebalancing Th1/Th2 immunity in AR [24]. For example, coptisine was shown to alleviate AR by suppressing mast cell degranulation and PI3K/Akt phosphorylation, leading to reduced levels of β-hexosaminidase, histamine, IL-4, and tumor necrosis factor (TNF)-α, as well as improved nasal symptoms and serum IgE levels in ovalbumin (OVA)-induced AR mice [25]. Tanshinone IIA mitigated allergic inflammation via inhibition of the PLCγ1/PKC/IP3R pathway, thereby reducing IgE-dependent histamine release and pro-inflammatory cytokines (TNF-α, IL-1β, IL-4, IL-5) in both RBL-2H3 cells and OVA-induced AR mice [26,27]. Similarly, essential oil derived from *Centipeda minima* was found to ameliorate AR by modulating TNF-α, IL-4, IgE, IL-2 and PTGS2/MAPK3 expression through multi-pathway involvement including neuroactive ligand-receptor interactions and Th17 cell differentiation [19]. Despite these promising findings, the specific bioactive compounds and mechanisms underlying the anti-AR effects of *Centipeda minima*—especially the differential roles of CMF and CMD—have not been fully elucidated. Furthermore, the integration of multi-omics technologies (genomics, epigenomics, transcriptomics, proteomics, metabolomics and microbiomics) has provided new opportunities for systematic investigation of AR pathogenesis and herbal medicine efficacy through comprehensive mapping of molecular signatures and biological networks [28,29].

In this study, we employed an OVA-induced murine AR model to evaluate and compare the therapeutic effects of CMF and CMD. Our methodology incorporated behavioral assessments, histopathological evaluation, and molecular analyses (ELISA and qRT-PCR), complemented by transcriptome sequencing to identify differentially expressed genes (DEGs). We further utilized widely targeted metabolomics and network pharmacology to identify active constituents, followed by in vitro validation in RBL-2H3 and RAW264.7 cells. Our work not only elucidates the pharmacodynamic distinctions between CMF and CMD at multiple biological levels but also provides molecular evidence supporting the traditional use of *Centipeda minima*. These findings offer novel mechanistic insights into the anti-allergic properties of this herb and facilitate its sustainable development and pharmaceutical application through science-based validation of bioactive compounds.

## 2. Results

### 2.1. Therapeutic Effect of CMF and CMD on AR Mice

**Figure 1:**
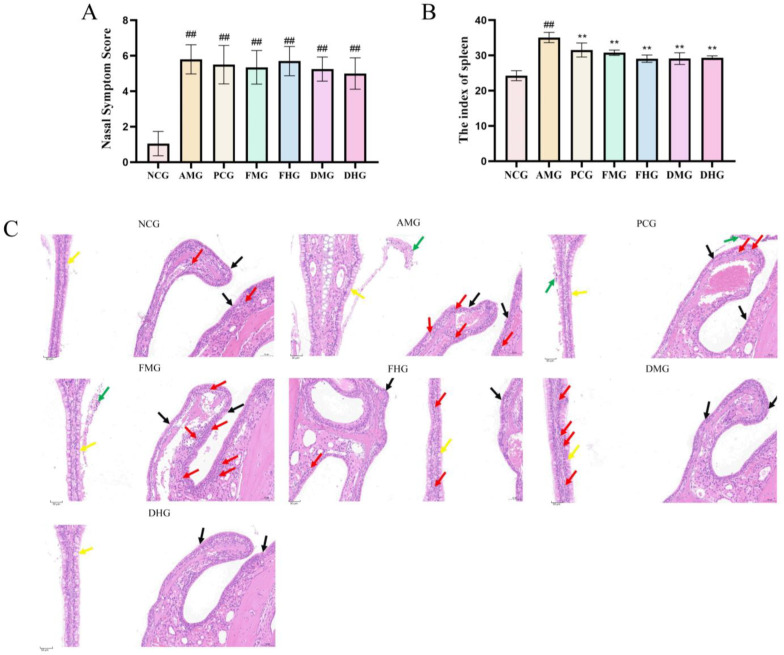
Phenotypic characterization of allergic rhinitis mouse model. (**A**) Mouse Nasal Symptoms Scoring of AR model before drug treatment (*n* = 10); (**B**) The effect of spleen index in mice after drug treatment (*n* = 10); (**C**) Histopathological changes in nasal mucosa in each group (H&E staining, 200×, the scale bar for this panel is 50 μm). Black arrows: ciliary loss; yellow arrows: goblet cells; red arrows: inflammatory cell infiltration; green arrows: epithelial shedding with hemorrhage. NCG shows intact architecture with minimal findings (inflammatory cell density: 18 cells/mm^2^). AMG exhibits severe pathological changes, including: marked inflammatory cell infiltration (red arrows, 78 cells/mm^2^), prominent goblet cell hyperplasia (yellow arrows), and focal epithelial shedding with hemorrhage (green arrows). Treatment Groups (PCG, FHG, DMG, DHG): Show varying degrees of improvement (inflammatory cell density: 16, 8, 18, 4 cells/mm^2^). Note the pronounced infiltration in FMG (inflammatory cell density: 94 cells/mm^2^); NCG: Negative Control group; AMG: AR Model group; PCG: Positive Control group; FMG: CMF Medium dose group; FHG: CMF High dose group; DMG: CMD Medium dose group; DHG: CMD High dose group; in contrast to the control group, ^##^ *p* < 0.01; In contrast to the model group, ** *p* < 0.01.

Within 30 min post-final nasal challenge, sensitized mice exhibited significantly increased nasal scratching, sneezing, and rhinorrhea compared to the blank control group (*p* < 0.05, Figure 1A). Behavioral scoring confirmed successful AR model establishment, with sensitized group scores exceeding 5 points versus controls (*p* < 0.05, Figure 1B). These behavioral manifestations closely mirrored clinical AR symptoms in humans, validating the model’s translational relevance. Surprisingly, CMF and CMD—treated group exhibited exacerbated sneezing episodes, suggesting a potential hypersensitivity reaction to the intervention.

HE staining revealed marked nasal mucosa alterations in model mice (Figure 1C). The nasal mucosa in the NCG maintained normal histological architecture, characterized by an intact epithelial layer with sparsely and evenly distributed goblet cells. The underlying lamina propria exhibited well-organized connective tissue fibers without signs of hyperplasia or inflammatory cell infiltration. In marked contrast, AMG demonstrated significant pathological alterations, including disorganized proliferation of goblet cells, pronounced vascular congestion, and extensive infiltration of inflammatory cells, particularly lymphocytes and eosinophils. All treatment groups including PCG, FHG, DMG and DHG showed substantial histological improvement, with restoration of normal connective tissue arrangement in the lamina propria. However, residual mild pathological changes persisted, consisting of focal glandular hyperplasia, localized vascular congestion, and minimal inflammatory cell infiltration, indicating partial but incomplete mucosal recovery following therapeutic intervention. These histological findings correlate well with the observed clinical improvements in each treatment group.

**Figure 2:**
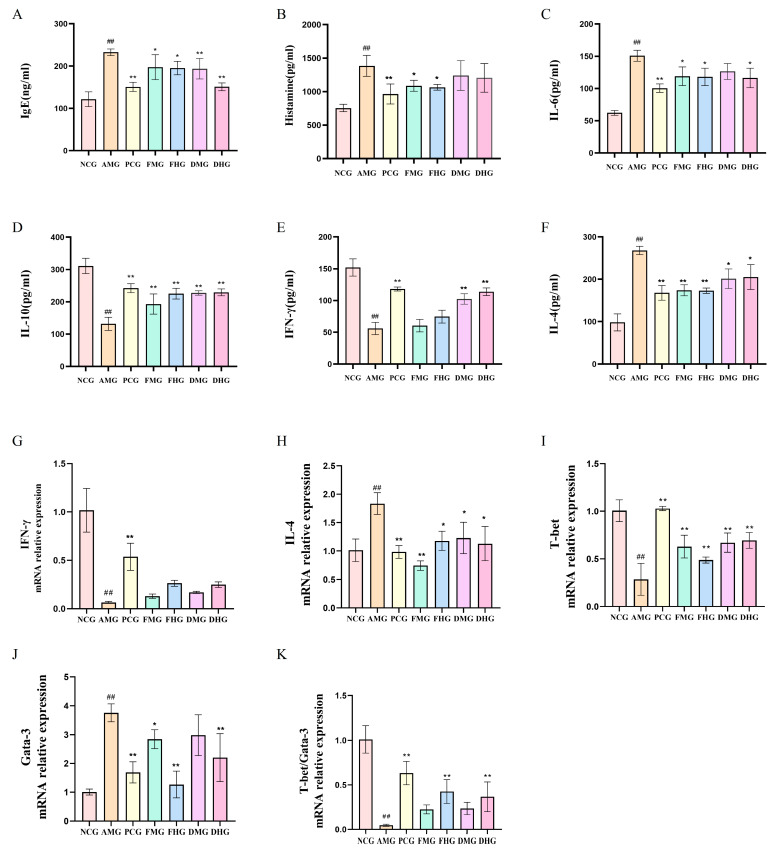
Serum levels and mRNA expression in each group (*n* = 10). (**A**) Serum levels of IgE; (**B**) Serum levels of HIS; (**C**) Serum levels of IL-6; (**D**) Serum levels of IL-10; (**E**) Serum levels of IFN-γ; (**F**) Serum levels of IL-4; (**G**) mRNA relative expression of IFN-γ; (**H**) mRNA relative expression of IL-4; (**I**) mRNA relative expression of T-bet; (**J**) mRNA relative expression of GATA-3; (**K**) mRNA relative expression of T-bet/GATA-3. NCG: Negative Control group; AMG: AR Model group; PCG: Positive Control group; FMG: CMF Medium dose group; FHG: CMF High dose group; DMG: CMD Medium dose group; DHG: CMD High dose group; in contrast to the control group, ^##^ *p* < 0.01; In contrast to the model group, * *p* < 0.05, ** *p* < 0.01.

The AR model group demonstrated characteristic Th2-skewed responses: elevated serum IgE, HIS, IL-6, and IL-4 (*p* < 0.01, Figure 2A–C,F), alongside suppressed IL-10 and IFN-γ levels (*p* < 0.01, Figure 2D,E). CMF treatment significantly reduced all pro-inflammatory markers (*p* < 0.05) while enhancing IL-10. CMD showed preferential upregulation of IFN-γ (*p* < 0.01, Figure 2E), indicating differential immunomodulatory mechanisms between fresh and dried preparations.

As shown in Figure 2G–K, the mice splenic mRNA expression revealed distinct Th1/Th2 immune dysregulation in the AR model, characterized by significant suppression of Th1-associated IFN-γ and T-bet expression alongside marked upregulation of Th2-related IL-4 and GATA-3 compared to controls (all *p* < 0.01, Figure 2H,J). While pharmacological treatments (FMG, MHG, DMG and DHG) failed to significantly modulate IFN-γ levels (*p* > 0.05, Figure 2G), they effectively attenuated Th2 polarization as evidenced by substantial IL-4 reduction (*p* < 0.05, Figure 2H). Both CMF and CMD interventions restored immune balance through differential mechanisms-all treatment groups significantly rescued T-bet expression (*p* < 0.01, Figure 2I) with CMD demonstrating superior Th1-promoting capacity, while high-dose preparations of both formulations significantly suppressed pathological GATA-3 overexpression (*p* < 0.01, Figure 2J). These molecular findings demonstrate that while CMF and CMD share the ability to rebalance Th1/Th2 immunity in AR, their therapeutic mechanisms diverge, with CMD preferentially enhancing Th1 responses and CMF more potently suppressing Th2 activity.

### 2.2. Splenic Transcriptome Profiling of Differentially Expressed Genes in AR Mice

Eukaryotic transcriptome sequencing (RNA-seq) of 21 samples generated 143.33 Gb of high-quality clean data, with each sample yielding ≥ 5.96 Gb and Q30 scores ≥ 93.72%. Clean reads aligned to the reference genome at efficiencies of 92.90–96.46%, enabling comprehensive analyses including alternative splicing prediction, gene structure optimization, and novel gene identification, which revealed 6265 novel genes (1580 functionally annotated). Using thresholds of |Fold Change| ≥ 1.5 and FDR < 0.01, differentially expressed genes (DEGs) were identified across all comparison groups, followed by functional enrichment (GO/KEGG).

**Figure 3:**
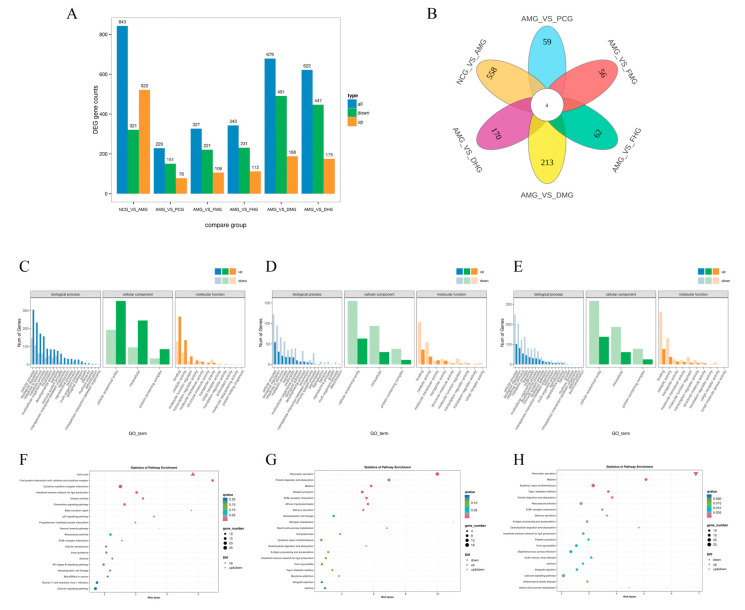
Transcriptome analysis of the spleen in mice (*n* = 3). (**A**) Bar chart for the comparison of DEGs among each group. (**B**) Venn diagram of overlapping DEGs across comparison groups. (**C**) GO classification of NCG vs. AMG; (**D**) GO classification of AMG vs. FHG; (**E**) GO classification of AMG vs. DMG; (**F**) KEGG pathway enrichdotplot of NCG vs. AMG; (**G**) KEGG pathway enrichdotplot of AMG vs. FHG; (**H**) KEGG pathway enrichdotplot of AMG vs. DMG. NCG: Negative Control group; AMG: AR Model group; PCG: Positive Control group; FMG: CMF Medium dose group; FHG: CMF High dose group; DMG: CMD Medium dose group; DHG: CMD High dose group.

Transcriptomic analysis revealed distinct patterns of differentially expressed genes (DEGs) across comparison groups (Figure 3A). The NCG vs. AMG set showed the highest total DEGs (843), with a marked up-regulation bias (522 up vs. 321 down). In contrast, all AMG vs. Others comparisons (AMG vs. PCG to AMG vs. DHG) exhibited consistent down-regulation dominance, particularly pronounced in AMG vs. DMG (491 down/188 up) and AMG vs. DHG (447 down/175 up). The AMG vs. PCG group had the fewest DEGs (229), while AMG vs. DMG and AMG vs. DHG displayed the most substantial transcriptional alterations (679 and 622 DEGs, respectively). These findings suggest AMG induces broad gene suppression relative to other conditions, whereas CMD triggers more balanced activation/repression responses.

To systematically compare the differences in gene expression profiles among experimental groups, Venn diagram analysis was performed, and the results revealed distinct patterns of shared and unique DEGs (Figure 3B). Four genes, Klf1, Adam1a, NewGene_6833, and NewGene_8321, were consistently differentially expressed across all six comparison groups, indicating their central role in the therapeutic response. For CMF treatments, the high-dose CMF (FHG) group shared 82 unique DEGs (e.g., Npy, Slc6a4, Camp) with other CMF groups, predominantly involved in neuro-immune modulation pathways. In CMD treatments, the CMD groups (DMG/DHG) exhibited 108 shared DEGs (e.g., Tlr9, Klf9, mt-Atp8), enriched in mitochondrial function and lipid metabolism. Regarding dose-dependent effects, the number of unique DEGs increased with the CMF dosage (from 327 in group FMG to 343 in group FHG), while CMD showed more extensive transcriptional changes (from 491 to 622 DEGs in groups DMG and DHG, respectively), with the high-dose CMD specifically upregulating Fbxl2, a key immune regulator. The PCG group treated with triamcinolone shared only 59 DEGs with other interventions, including Bbc3 related to apoptosis and mt-Nd1 associated with oxidative phosphorylation, reflecting its distinct pharmacological mechanism.

The comprehensive GO enrichment analysis of six comparison groups revealed distinct functional patterns in allergic rhinitis pathogenesis and treatment responses (Figure 3C–E). The NCG vs. AMG comparison (704 DEGs) showed the strongest enrichment in immune system processes (75 genes, 10.7%) and inflammatory responses (234 genes in “response to stimulus”), with predominant intracellular localization (340 genes, 48.3%) and binding functions (395 genes, 56.1%), confirming the robust immunological basis of AR. The positive control group (AMG vs. PCG, 190 DEGs) exhibited the most focused effects, with significant reductions in immune-related terms (only 14 genes in immune system processes) but maintained catalytic activity (49 genes, 25.8%), suggesting targeted pharmacological action. Both fresh *C. minima* interventions (AMG vs. FMG and AMG vs. FHG) demonstrated intermediate-scale transcriptional changes (263 and 274 DEGs, respectively), showing partial normalization of AR signatures, particularly in metabolic processes (83 and 74 genes) with relatively preserved immune modulation (22 and 28 immune-related genes). Notably, the dried *C. minima* treatments (AMG vs. DMG and AMG vs. DHG) displayed broader transcriptional impacts (582 and 503 DEGs), with the strongest metabolic reprogramming (183 and 162 metabolic process genes) coupled with sustained immune regulation (54 and 47 immune system genes), while uniquely maintaining molecular transducer activity (34 and 38 genes). All treatment groups showed complete loss of antioxidant activity genes present in NCG vs. AMG, suggesting alternative oxidative stress management mechanisms. The dried preparations particularly influenced developmental processes (91 and 90 genes) and cellular signaling (117 and 107 genes), indicating their potential for tissue repair and broader pathway modulation compared to the more focused effects of fresh preparations and the positive control. These results collectively illustrate a spectrum of therapeutic mechanisms, from the targeted immunosuppression of PCG to the comprehensive immune-metabolic regulation by CMD, with CMF occupying an intermediate position.

The KEGG pathway enrichment analysis comparing six different gene groups (NCG vs. AMG, AMG vs. PCG, AMG vs. FMG, AMG vs. FHG, AMG vs. DMG, and AMG vs. DHG) revealed several key biological insights (Figure 3F–H). Highly enriched pathways with significant *p*-values and q-values (q < 0.05) included Pancreatic secretion (enrich_factor = 9.98, *p* = 8.51 × 10^−13^, q = 1.57 × 10^−10^ in AMG vs. FMG), Nitrogen metabolism (enrich_factor = 11.48, *p* = 0.002, q = 0.142 in AMG vs. FHG), and Malaria (enrich_factor = 5.42, *p* = 0.0001, q = 0.0206 in AMG vs. FHG), suggesting strong metabolic and immune-related adaptations. The cell cycle pathway was notably enriched in NCG vs. AMG (enrich_factor = 4.83, *p* = 7.53 × 10^−10^, q = 1.94 × 10^−7^), indicating potential differences in cellular proliferation. Common pathways across multiple comparisons, such as Protein digestion and absorption, Type I diabetes mellitus, and Systemic lupus erythematosus, highlighted recurring themes in metabolism and immune regulation. Immune-related pathways like Cytokine-cytokine receptor interaction and Complement and coagulation cascades were frequently observed, implying a consistent role of immune responses across comparisons. Digestive system pathways, including Fat digestion and absorption and Carbohydrate digestion and absorption, were also prominent, possibly reflecting nutrient-processing mechanisms. While many pathways exhibited low *p*-values, only a subset remained significant after multiple testing correction (q < 0.05), emphasizing the importance of stringent statistical thresholds. Overall, the results suggest that metabolic, immune, and digestive pathways play central roles in the functional distinctions between these gene groups, with some pathways consistently enriched across different comparisons, pointing to shared biological mechanisms.

**Figure 4:**
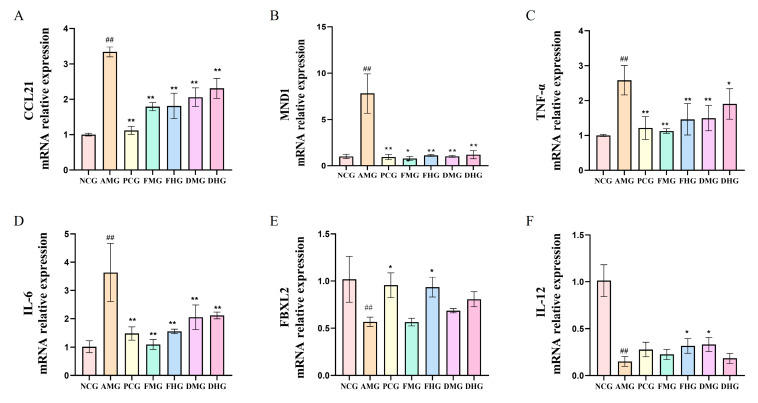
DEGs validation by qRT-PCR (*n* = 3). (**A**) mRNA relative expression of *CCL21*; (**B**) mRNA relative expression of *MND1*; (**C**) mRNA relative expression of *TNF-α*; (**D**) mRNA relative expression of *IL-6*; (**E**) mRNA relative expression of FBXL2; (**F**) mRNA relative expression of *IL-12*. NCG: Negative Control group; AMG: AR Model group; PCG: Positive Control group; FMG: CMF Medium dose group; FHG: CMF High dose group; DMG: CMD Medium dose group; DHG: CMD High dose group. In contrast to the control group, ^##^ *p* < 0.01; In contrast to the model group, * *p* < 0.05, ** *p* < 0.01.

**Figure 5:**
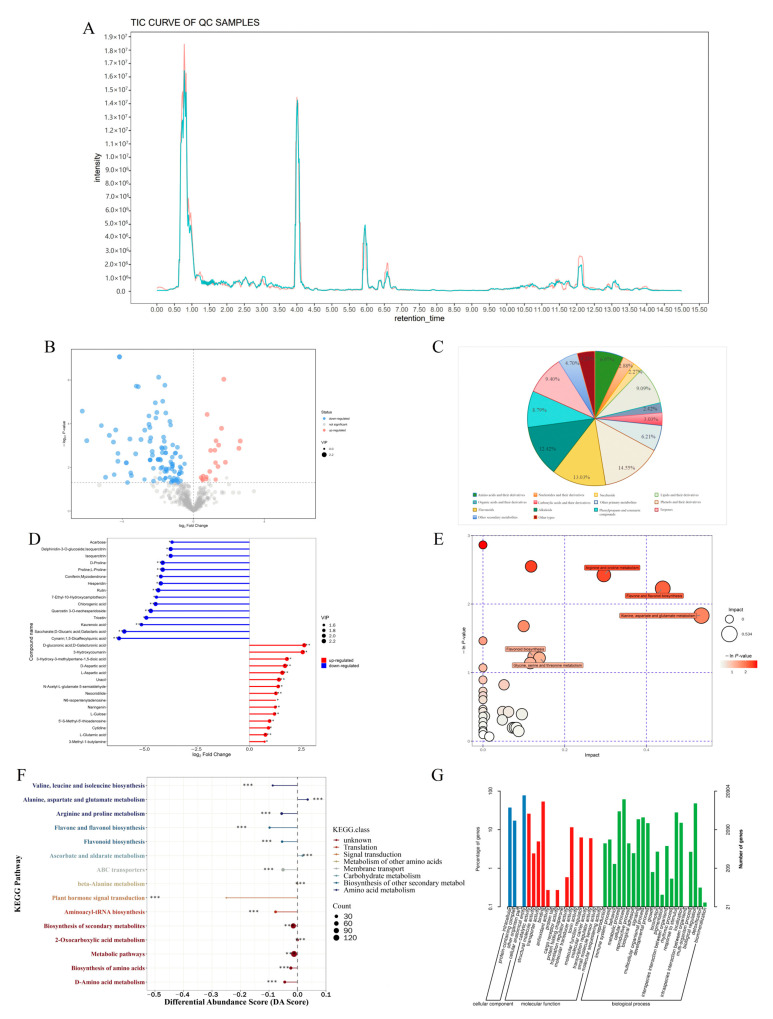
Differential metabolites analysis in CMF and CMD (*n* = 6). (**A**) Total Ion Chromatogram (TIC); (**B**) volcano plot for screening differential metabolites between CMF and CMD; (**C**) metabolite profiling of *Centipeda minima*; (**D**) matchstick plot of differential metabolites; (**E**) bubble plot of pathway analysis; (**F**) KEGG DA score plot of enrichment analysis; (**G**) GO enrichment analysis. CMF vs. CMD: * indicates *p* < 0.05, ** indicates *p* < 0.01, and *** indicates *p* < 0.001.

Six key genes closely associated with immune-inflammatory responses were selected for further investigation (Figure 4). qRT-PCR validation demonstrated that the AMG exhibited significantly elevated expression levels of pro-inflammatory factors (*CCL21*, *MND1*, *TNF-α* and *IL-6*) compared with NCG (*p* < 0.01, Figure 5A–D), while the expression levels of anti-inflammatory related genes *FBXL2* and *IL-12* was markedly downregulated (*p* < 0.01, Figure 5E,F). Following therapeutic intervention, both CMF and CMD exhibited significant anti-inflammatory effects: medium- and high-dose CMF significantly suppressed the overexpression of *CCL21*, *IL-6*, *TNF-α* (*p* < 0.01,Figure 5A,C,D) and *MND1* (*p* < 0.05, Figure 5B), with high-dose CMF notably upregulating FBXL2 and IL-12 expression levels (*p* < 0.05, Figure 5E,F). Similarly, medium- and high-dose CMD treatment also effectively downregulated these pro-inflammatory factors (*p* < 0.01 or *p* < 0.05, Figure 5A–D), with medium-dose CMD specifically upregulating *IL-12* expression (*p* < 0.05, Figure 5F). These results not only validated the reliability of transcriptomic data but also revealed the molecular mechanisms by which CMF and CMD exert therapeutic effects through differential regulation of key immune-inflammatory related gene expression.

### 2.3. Differential Metabolites Identification in CMF and CMD

The chromatographic analysis revealed excellent instrument stability, as evidenced by consistent retention times and highly reproducible peak profiles in the total ion chromatograms (Figure 5A). Comparative analysis revealed 121 significantly altered metabolites between CMF and CMD, with 20 upregulated and 101 downregulated compounds in CMD relative to CMF (Figure 5B). Global metabolomic profiling identified 660 compounds across all samples, which were systematically classified into 14 metabolic categories (Figure 5C). Primary metabolites accounted for 32.87% of detected compounds, including amino acid derivatives (6.97%), nucleotides (2.88%), carbohydrates (2.27%), and lipids (9.09%). Secondary metabolites represented 62.69% of the metabolome, predominantly phenolics (14.55%), flavonoids (13.03%), alkaloids (12.42%), phenylpropanoids (8.79%), and terpenoids (9.40%).

Notably, fresh samples showed higher accumulation of naringenin and cnicin, while dried specimens exhibited elevated levels of various flavonoids (quercetin-3-O-neohesperidoside, rutin, hyperoside) and phenolic compounds (ethyl vanillin, gentisic acid, 6-gingerol) (Figure 5D, Table 1). The GO and KEGG enrichment analysis showed that there are profound alterations in amino acid metabolism, phenolic/flavonoid pathways, and carbohydrate derivatives between CMF and CMD (Figure 5E–G). These distinct metabolic profiles suggest substantial phytochemical modifications during the drying process.

### 2.4. Network Pharmacological Analysis of Differential Metabolites in AR Activity

Through systematic pharmacological investigation, we identified 121 differential metabolites between CMF and CMD, of which 114 bioactive compounds were predicted to target 964 potential proteins. Integration of allergic rhinitis-associated targets from Genecards and DisGeNET databases yielded 2388 disease-related targets, with Venn analysis revealing 256 overlapping targets (Figure S1). Protein–protein interaction (PPI) network analysis highlighted 10 core targets, including TNF, IL6, and IL10 (Table 2), while the compound-target-disease network identified five key bioactive constituents (Figure S2): neocnidilide and naringenin (enriched in CMF), along with 6-gingerol, 2′,3,5,7-tetrahydroxyflavone, and clemastanin A (predominant in CMD). Based on their high variable importance in projection (VIP) scores (Table 1) and target connectivity, neocnidilide (a fresh sample-specific metabolite, VIP: 1.8927) and 6-gingerol (a drying-derived component, VIP: 2.1281) were selected for further pharmacological validation.

**Figure 6:**
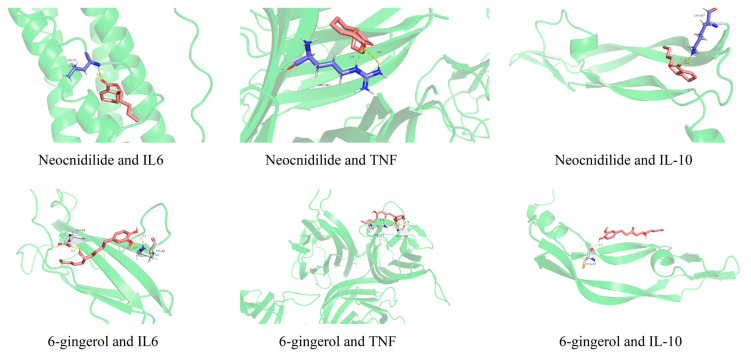
Molecular docking diagram of neocnidilide and 6-gingerol with IL-6, TNF, and IL-10.

Functional enrichment analysis demonstrated significant associations with inflammatory response and cell proliferation regulation (Figure S3), particularly involving the AGE-RAGE and HIF-1 signaling pathways (Figure S4). Notably, Th17 cell differentiation and PD-1/PD-L1 checkpoint pathways were implicated, suggesting immunomodulatory mechanisms underlying the anti-allergic effects. Molecular docking analysis revealed the binding affinities of the active components (neocnidilide and 6-gingerol) with key inflammatory targets (IL-6, TNF, and IL-10). As shown in Figure 6, neocnidilide exhibited strong binding energies of −4.71, −3.75, and −3.80 kcal/mol with IL-6, TNF, and IL-10, respectively. In contrast, 6-gingerol showed weaker interactions, with binding energies of −0.87, −0.59, and −0.35 kcal/mol for the same targets. These results suggest that neocnidilide may have a more potent binding affinity toward the core targets compared to 6-gingerol.

These findings systematically elucidate the transformation patterns of bioactive constituents during the drying process and reveal the multi-target, multi-pathway mechanisms of *Centipeda minima* against allergic rhinitis. The discovery that fresh sample-derived neocnidilide and processed herb-specific 6-gingerol may modulate divergent inflammatory networks offers a theoretical foundation for developing tailored formulations based on different processing methods.

### 2.5. Cellular Evidence for the Anti-Allergic and Anti-Inflammatory Properties of CMF and CMD Differential Metabolites

**Figure 7:**
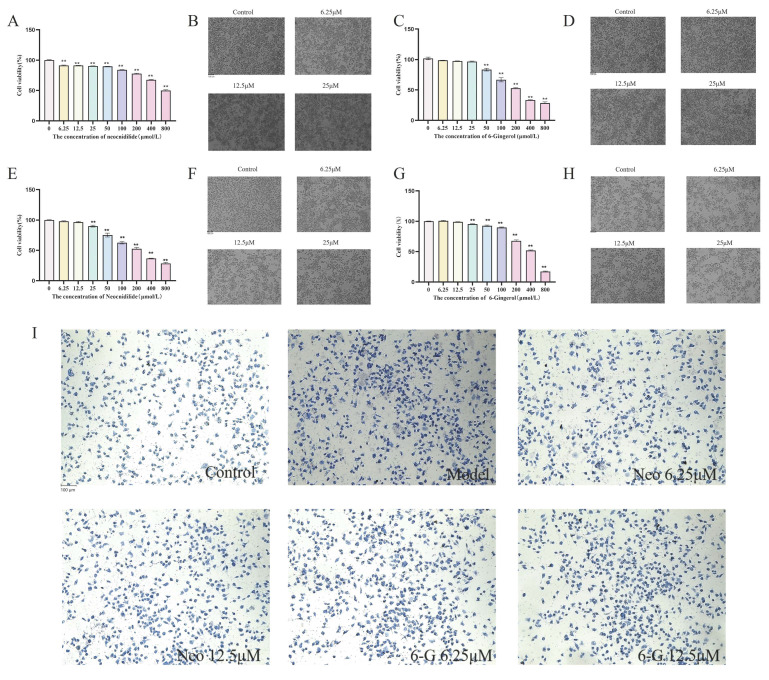
Cytotoxicity and effect of neocnidilide and 6-gingerol on RBL-2H3 and RAW264.7 cells (*n* = 3). (**A**,**B**) Effect of Neocnidilide on cell viability of RBL-2H3 (100×); (**C**,**D**) Effect of 6-Gingerol on cell viability of RBL-2H3 (100×); (**E**,**F**) Effect of Neocnidilide on cell viability of RAW264.7 (100×); (**G**,**H**) Effect of 6-Gingerol on cell viability of RAW264.7 (100×). (**I**) IgE-induced degranulation of RBL-2H3 cells stained with toluidine blue (100×). The scale bar for this panel is 100 μmIn contrast to the control group, ** *p* < 0.01.

The cytotoxic effects of neocnidilide (NEO) and 6-gingerol (6-G) were systematically evaluated in RBL-2H3 and RAW264.7 cell lines. Concentration-response analyses (6.25–800 μM) demonstrated dose-dependent reductions in cell viability (Figure 7A–H), with non-cytotoxic thresholds established at ≤12.5 μM for RBL-2H3 cells and ≤25 μM for RAW264.7 macrophages. These safety profiles guided subsequent experimental concentrations to ensure cellular integrity while maintaining pharmacological activity.

**Figure 8:**
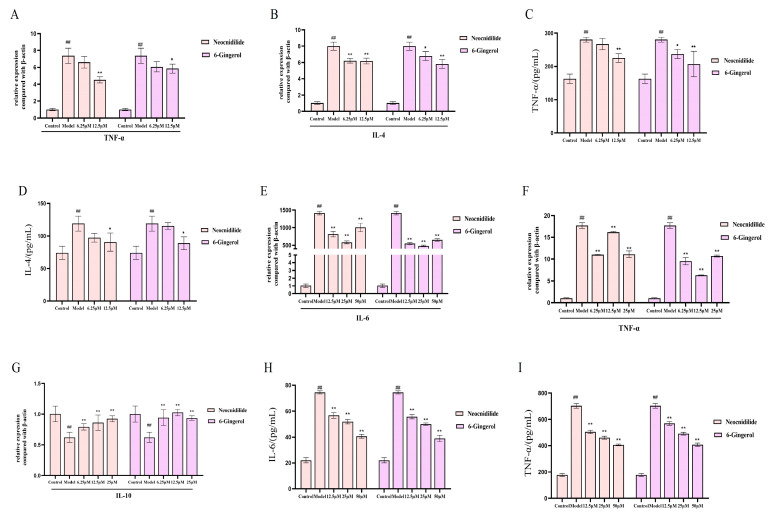
Anti-degranulation effects and cytokine modulation by NEO and 6-G in IgE-activated mast cells (*n* = 3). (**A**) The relative expression of *TNF-α* in RBL-2H3 cells; (**B**) the relative expression of *IL-4* in RBL-2H3 cells; (**C**) effect on TNF-α release in IgE-induced RBL-2H3 cell degranulation model; (**D**) effect on IL-4 release in IgE-induced RBL-2H3 cell degranulation model; (**E**) effect on mRNA expression of *IL-6* in RAW264.7 cells induced by LPS; (**F**) effect on mRNA expression of *TNF-α* in RAW264.7 cells induced by LPS; (**G**) effect on mRNA expression of IL-10 in RAW264.7 cells induced by LPS; (**H**) the levels of IL-6 in LPS-stimulated RAW264.7 cells culture supernatant; (**I**) the levels of TNF-α in LPS-stimulated RAW264.7 cells culture supernatant. In contrast to the control group, ^##^ *p* < 0.01; in contrast to the model group * *p* < 0.05, ** *p* < 0.01.

In IgE-sensitized RBL-2H3 cells, control groups maintained normal spindle morphology without degranulation, while model groups exhibited characteristic degranulation phenotypes including darkened staining, cellular shrinkage, and peripheral blue-purple granule release (arrow indicated). Treatment with NEO or 6-G (6.25–12.5 μM) effectively preserved cellular morphology (Figure 7I) and significantly attenuated degranulation markers (*p* < 0.01, Figure 8). qRT-PCR analysis revealed dose-dependent suppression of IgE-induced *TNF-α* (57.8–64.2%) and IL-4 (52.4–59.1%) mRNA overexpression (*p* < 0.01) (Figure 8A,B), with ELISA further confirming inhibition of corresponding cytokine secretion (*p* < 0.01, Figure 8C,D). For LPS-stimulated RAW264.7 macrophages, both compounds (25 μM) exhibited significant immunomodulatory effects by suppressing pro-inflammatory cytokines *IL-6* and *TNF-α* (*p* < 0.01, Figure 8E,F,H,I) while enhancing anti-inflammatory IL-10 production (*p* < 0.01, Figure 8G).

## 3. Discussion

Our study systematically validated the successful establishment of an ovalbumin-induced AR mouse model through comprehensive behavioral, histopathological and immunological assessments. The model animals exhibited characteristic allergic responses including significantly elevated nasal irritation scores, marked mucosal inflammation with eosinophil infiltration, and typical Th2 immune polarization, consistent with previous reports in AR models [30,31]. Notably, the observed dramatic increase in IgE and histamine levels, coupled with the characteristic cytokine imbalance (elevated IL-4 and suppressed IFN-γ), closely mirrors the immunopathological features of clinical AR [1,32]. However, it is important to critically acknowledge that while murine models provide valuable mechanistic insights, they cannot fully recapitulate the complex etiology and heterogeneous presentations of human AR due to species-specific differences in immune regulation and nasal architecture.

Through integrated pharmacological investigations, we have elucidated the distinct immunomodulatory mechanisms of CMF and CMD preparations in AR treatment. The differential regulation of Th1/Th2 balance observed in our study aligns well with current understandings of AR pathophysiology where Th2-dominant responses drive IgE-mediated inflammation [33,34,35]. CMF demonstrated superior suppression of Th2 cytokines including IL-4 and IL-6, along with significant downregulation of GATA-3 expression, which correlates with its higher content of sesquiterpenes, particularly neocnidilide. Our in vitro assays confirmed neocnidilide’s potent inhibition of STAT6 signaling, providing mechanistic insight that complements recent reports on sesquiterpenes’ immunomodulatory properties [36].

Transcriptomic profiling revealed 1123 differentially expressed genes between treatment groups, with CCL21 emerging as a novel therapeutic target. The elevated expression of this chemokine in AR models and its subsequent downregulation by CMF/CMD treatment suggests its pivotal role in eosinophil recruitment, corroborating emerging evidence of CCL21-CCR7 axis involvement in allergic inflammation [37,38]. Furthermore, the concurrent modulation of FBXL2, an E3 ubiquitin ligase known to negative regulation of NLRP3 inflammasome assembly [39], provides important mechanistic insight into CMF’s anti-inflammatory effects and extends recent discoveries regarding ubiquitin-proteasome system involvement in AR pathogenesis [40].

Metabolomic analysis coupled with network pharmacology identified 6-gingerol as a key active component in CMD, which exhibited remarkable Th1-promoting activity through specific upregulation of T-bet expression. This finding aligns with growing pharmacological interest in gingerol as natural immunomodulator [41,42], while explaining CMD’s unique ability to enhance IFN-γ production [43]. The predominance of flavonoids and phenolic compounds in CMD accounts for its significant antioxidant properties, providing support for the oxidative stress theory of AR pathogenesis [44].

Our cellular experiments using RBL-2H3 mast cells and RAW264.7 macrophages provided crucial mechanistic validation of the dual anti-allergic mechanisms: neocnidilide-mediated mast cell stabilization (evidenced by reduced histamine release) and 6-gingerol-induced macrophage polarization (demonstrated by enhanced IL-10 production). This multi-target action exemplifies the network pharmacology paradigm of herbal medicines, enabling more comprehensive modulation of AR’s complex pathogenesis compared to single-target synthetic drugs [45,46,47,48]. The dose-dependent upregulation of FBXL2 and IL-12 by high-dose CMF suggests potential epigenetic regulatory mechanisms that warrant further investigation into possible miRNA involvement [49,50].

The complementary Th1/Th2 modulation by CMF and CMD presents intriguing translational possibilities for clinical AR therapy. The fresh preparation’s dominance in mast cell stabilization and Th2 suppression suggests potential application in acute exacerbation phases, while the dried form’s strength in enhancing Th1 responses indicates potential for long-term immune regulation. However, several challenges must be addressed before clinical translation: First, the pharmacokinetic profiles and bioavailability of neocnidilide and 6-gingerol require thorough investigation in human subjects. Second, standardized extraction and quality control protocols must be established to ensure batch-to-batch consistency. Third, potential differences in human immune responses compared to murine models necessitate careful phase I clinical trials to assess safety and efficacy.

While our OVA-induced murine model provides valuable insights, several limitations should be considered: The model employs artificial sensitization with alum adjuvant, which does not fully mimic natural allergen exposure in humans; The short-term intervention paradigm (14 days) may not capture long-term adaptive immune changes; Species differences in drug metabolism and immune system components may affect translational predictability. Future studies should incorporate human nasal epithelial cell cultures and clinical samples to validate these findings. Additionally, exploring combination therapies integrating CMF/CMD with conventional treatments could reveal synergistic effects worthy of clinical development.

This study provides scientific validation for traditional processing methods while establishing evidence-based guidelines for differential application of fresh versus dried preparations. The identified component-target network offers potential quality control biomarkers for *Centipeda minima*, and the discovered complementary mechanisms lay foundation for developing synergistic formulations. Future research should focus on human clinical trials to fully establish the therapeutic potential of these preparations in allergic rhinitis management.

## 4. Material and Methods

### 4.1. Chemicals and Reagents

All chemicals and reagents utilized in this study were of analytical grade or higher purity, procured from certified suppliers. Ovalbumin (Shanghai Aladdin Biochemical Technology Co., Ltd., Shanghai, China) and aluminum hydroxide (Shanghai Solarbio Science & Technology Co., Ltd., Shanghai, China) were used for sensitization, with triamcinolone acetonide nasal spray (Kunming Yuanrui Pharmaceutical Co., Ltd., Kunming, China) as the positive control. Cytokine levels were measured using commercial mouse ELISA kits (Wuhan Bioswamp Biological Technology Co., Ltd., Wuhan, China), while histological analysis employed H&E staining reagents (Wuhan Servicebio Technology Co., Ltd., Wuhan, China) and standard solvents (Sigma-Aldrich Trading Co., Ltd., Shanghai, China or Sinopharm Group Co., Ltd., Shanghai, China).

### 4.2. Animals and Treatment

Male Balb/c mice (6–7 weeks old, SPF grade) were obtained from Shanghai Slack Laboratory Animal Co., Ltd., Shanghai, China (Animal Production License No.: SCXK (Hu) 2022-0004). The mice were housed in the SPF facility of Fujian Provincial Hospital Animal Center (License No.: SYXK (Min) 2023-0005) under specific pathogen-free conditions with controlled environmental conditions (temperature: 20 ± 2 °C; stable pressure; adequate ventilation). All animals had ad libitum access to standard rodent diet and sterile water, with daily cage maintenance performed by trained personnel to ensure hygienic conditions.

Fresh *Centipeda minima* (*Asteraceae*) plants were collected from Baiquan Village (24.0302° N, 117.4057° E), Fujian Province, China, with fresh juice (CMF) prepared by mechanical homogenization and volumetric standardization, while dried material (CMD) was obtained through shade-drying and traditional decoction. Based on human-mouse dose conversion, two therapeutic concentrations (650 and 1300 mg/kg) were prepared for both CMF and CMD, with triamcinolone acetonide nasal spray (1.1 mg/mL) serving as positive control—all sterilized aliquots were stored at 4 °C until use. Voucher specimen (#20220424) was deposited in Fujian Key Laboratory of Medical Analysis, Fujian Academy of Medical Sciences.

**Figure 9:**
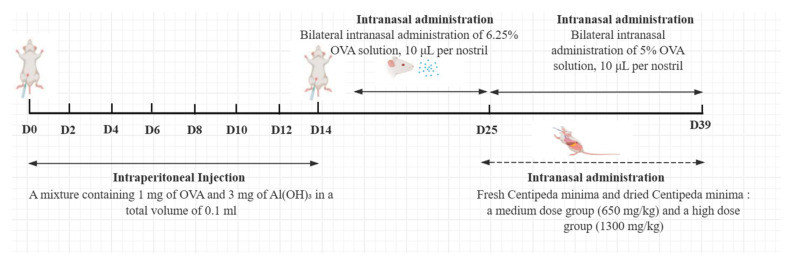
Timeline of AR mouse modeling and treatment.

Seventy SPF-grade male Balb/c mice were randomly assigned to seven experimental groups (*n* = 10 per group) using a computer-generated random number sequence: (A) Negative control group (NCG, PBS treated), (B) AR model (AMG, OVA-sensitized), (C) positive control (PCG, 33 μg/kg triamcinolone acetonide treated), (D) CMF medium-dose (FMG, 650 mg/kg CMF treated), (E) CMF high-dose (FHG, 1300 mg/kg CMF treated), (F) CMD medium-dose (DMG, 650 mg/kg CMD treated), and (G) CMD high-dose (DHG, 1300 mg/kg CMD treated). All treatments were administered intranasally (20 μL/dose) twice daily at 8 h intervals for 14 consecutive days. Dose selection was based on preliminary experiments and human-mouse equivalent dose conversion. Researchers performing animal handling and outcome assessments were blinded to group assignments throughout the experiment. A schematic overview of the experimental design is provided in Figure 9.

### 4.3. General Behavioral Scoring

Throughout the modeling period, mice were monitored for general health status including activity level, food/water intake, fur condition, and nasal symptoms. Following the final nasal challenge, AR-specific behaviors (nose scratching, sneezing, and clear nasal discharge) were quantitatively assessed during a standardized 15 min observation window. Model success was confirmed when animals exhibited composite behavioral scores exceeding 5 points according to established criteria, with concurrent observation of characteristic allergic manifestations such as peri-ocular rubbing and nasal inflammation. The severity of allergic rhinitis symptoms in mice was evaluated using a standardized scoring system. Nasal itching was scored as follows: 1 point for mild scratching (1–2 episodes), 2 points for moderate scratching (intermediate frequency), and 3 points for severe scratching (repeated episodes). Sneezing was graded as 1 point (1–3 sneezes), 2 points (4–10 sneezes), or 3 points (>11 sneezes). Rhinorrhea was assessed based on nasal discharge: 1 point for moist nostrils, 2 points for discharge around the nostrils, and 3 points for discharge extending beyond the nostrils. The total symptom score was calculated by summing the individual scores for each parameter.

### 4.4. Murine Spleen Index Analysis

At the experimental endpoint, all mice were euthanized and their spleens were carefully excised. The organs were blotted dry on filter paper to remove residual blood before precise weighing (analytical balance ± 0.1 mg). Spleen indices were calculated using the standardized formula: Spleen Index (mg/10 g body weight) = [Spleen Weight (mg)]/[Body Weight (g)] × 10.

### 4.5. Serum Biochemical Analysis

Twenty-four hours after the final allergen challenge, blood samples were obtained via retro-orbital puncture under mild isoflurane anesthesia (2%). Using sterile ophthalmic forceps, the eyeball was gently protruded to allow passive blood flow into pre-chilled 1.5 mL Eppendorf tubes. Following complete exsanguination, mice were humanely euthanized by cervical dislocation. The collected blood samples were allowed to clot at room temperature for 2 h, followed by centrifugation at 3000× *g* for 15 min at 4 °C. The resulting serum supernatant was carefully aliquoted using low-retention pipette tips and stored at −80 °C until subsequent biochemical analyses (IgE, HIS, IL-4, IL-6, IL-10, and IFN-γ measurements by ELISA).

### 4.6. Histopathological Examination

Nasal mucosa specimens were collected from three mice per group, fixed in 4% paraformaldehyde, and processed through standardized paraffin embedding with gradient ethanol dehydration and xylene clearing. Serial 4 μm tissue sections were routinely stained with hematoxylin and eosin (H & E) using optimized protocols (5 min hematoxylin, 15 s eosin) to evaluate epithelial integrity, inflammatory cell infiltration, and mucosal remodeling. All slides were digitally scanned by PANNORAMIC whole slide scanner (3DHISTECH, Budapest, Hungary).

### 4.7. Transcriptome Analysis

Total RNA was extracted from flash-frozen nasal mucosa tissues (*n* = 3/group) using TRIzol reagent, with RNA integrity verified (RIN ≥ 8.0) by Agilent 2100 Bioanalyzer. Libraries were prepared following the NEBNext Ultra II protocol, including poly (A) selection, fragmentation, and 150 bp paired-end sequencing on an Illumina NovaSeq 6000 platform (Novogene, Beijing, China). Raw sequencing data were processed through the standardized bioinformatics pipeline (BMKCloud Platform, Biomarker Technologies, Beijing, China). Raw reads were aligned to GRCm38 genome (release 95) using HISAT2(v 2.0.4), with gene expression quantified via StringTie (v2.2.1, RPKM values). Sample reproducibility was verified through Pearson correlation analysis (r^2^ > 0.9). Differential expression analysis was performed using DESeq2 (v1.30.1, |log_2_FC| > 1, FDR < 0.05), followed by functional enrichment (GO/KEGG) and protein interaction network analysis (STRING). Significantly enriched pathways were identified based on q-values (FDR-adjusted *p*-values) < 0.05. Six key DEGs were validated by RT-qPCR.

### 4.8. Widely Targeted Metabonomic Analysis

Lyophilized *Centipeda minima* samples weighing 50 mg were homogenized in 700 μL of an ice-cold methanol–water mixture with a ratio of 3:1 that contained 2-chlorophenylalanine as the internal standard. The extraction process involved vortex mixing, mechanical homogenization at 35 Hz for 4 min, and ice-bath ultrasonication for 5 min, repeating these steps for three cycles. Then, it was shaken at 4 °C for 14–16 h and centrifuged at 12,000 rpm for 15 min. The supernatant was 0.22 μm-filtered before further analysis, and for quality control samples, it was diluted 20-fold before being stored at −80 °C. Chromatographic separation was achieved using a Waters Acquity UPLC HSS T3 column with a particle size of 1.8 μm and dimensions of 2.1 × 100 mm. A gradient elution was employed using 0.1% (*v*/*v*) aqueous formic acid solution and acetonitrile, where the acetonitrile concentration increased from 2% to 95% over the period of 0.5 to 13.1 min at a constant flow rate of 400 μL/min with the column temperature maintained at 40 °C. Mass spectrometric detection was conducted on a SCIEX 6500 QTRAP+ system with specific ESI source parameters: an ion spray voltage of ±5500/4500 V, ion source operated at 400 °C with 35 psi curtain gas, GS1/GS2 gas pressures of 60 psi, and a declustering potential of ±100 V in the MRM mode. The raw data were processed by Biotree (Shanghai, China) with the SCIEX Analyst software (v1.6.3) for peak identification, quantification, and quality control.

Principal Component Analysis (PCA) was used for unsupervised pattern recognition and to assess overall metabolic variability between groups. Orthogonal Partial Least Squares-Discriminant Analysis (OPLS-DA) was applied for supervised modeling to maximize separation between groups and identify metabolites contributing most to class discrimination. Metabolites with Variable Importance in Projection (VIP) > 1.0 (from OPLS-DA) and *p* < 0.05 (from *t*-test) were considered significantly altered. Metabolites were identified by matching exact mass and MS/MS fragmentation patterns against HMDB, KEGG, and self-built databases. Integrative analysis was performed using Spearman correlation.

### 4.9. Network Pharmacology and Molecular Docking Analysis

A comprehensive network pharmacology approach was implemented to elucidate the therapeutic mechanisms of *Centipeda minima*. First, SMILES structures of differential metabolites from CMF and CMD were obtained via PubChem and imported into Swiss Target Prediction to identify potential targets. Disease-associated targets for allergic rhinitis (AR) were retrieved from GeneCards and DisGeNET databases. Using Venny 2.1.0, shared targets between metabolites and AR were identified and subsequently analyzed in STRING(v12.0) to construct protein–protein interaction (PPI) networks, visualized using Cytoscape 3.7.2. Functional enrichment analysis was performed via DAVID to identify key GO terms (top 20 each for biological processes, cellular components, and molecular functions) and KEGG pathways (top 20). Molecular docking was conducted by obtaining target protein structures from PDB and ligand structures from PubChem, with binding analyses performed using AutoDock Vina (v1.2.0) Tools and visualized in PyMOL (v2.1.0).

### 4.10. Cells and Treatment

RBL-2H3 and RAW264.7 cells were exposed to varying concentrations (6.25, 12.5, 25, 50, 100, 200, 400, 800 μM) of Neocnidilide (NEO) and 6-Gingerol (6-G). Cell viability was assessed using the CCK-8 assay, while morphological alterations of both cell lines were visualized via inverted microscopy (Leica DMI8, Wetzlar, Germany). Subsequently, gene expression levels of *TNF-α*, *IL-6*, *IL-10*, and *IL-4* were quantified by qRT-PCR, while corresponding TNF-α, IL-6, and IL-4 protein concentrations were measured by ELISA.

### 4.11. Statistical Analysis

All statistical analyses were performed using GraphPad Prism version 8.0 (GraphPad Software, San Diego, CA, USA). For comparisons involving more than two groups, one-way analysis of variance (One-way ANOVA) was employed. A *p*-value less than 0.05 was considered to indicate that the difference was statistically significant. Student’s t-test was employed to identify significant differences in metabolite levels between comparison groups, with a significance threshold of * *p* < 0.05.

## 5. Conclusions

This study reveals the distinct yet complementary mechanisms of fresh and dried *Centipeda minima* in alleviating AR, with CMF suppressing Th2-mediated inflammation and CMD enhancing Th1 responses through modulation of NF-κB and JAK-STAT signaling pathways. Integrated metabolomic and transcriptomic analyses identified neocnidilide and 6-gingerol as key bioactive compounds responsible for these immunomodulatory effects, while also uncovering critical regulatory genes associated with immune polarization. The differential chemical profiles—CMF rich in amino acids/nucleotides and CMD abundant in flavonoids/phenols—provide a scientific basis for their traditional applications and establish quality control markers for standardized preparations. These findings not only bridge ethnopharmacological knowledge with modern molecular mechanisms but also highlight the potential of *Centipeda minima* as a source of targeted bioactive compounds for developing precision therapies against AR and related inflammatory diseases.

## Figures and Tables

**Table 1:** The top 30 differential metabolites with the highest fold changes.

NO.	Compound	Category	Formula	VIP	Trend
1	D-Aspartic acid	Alkaloids	C_4_H_7_NO_4_	2.1657	↑
2	3-Hydroxycoumarin	Coumarins	C_9_H_6_O_3_	2.1513	↑
3	L-Aspartic acid	Amino acids	C_4_H_7_NO_4_	2.1313	↑
4	D-glucoronic acid	Carbohydrates	C_6_H_10_O_7_	2.0943	↑
5	Uracil	Nucleotide	C_4_H_4_N_2_O_2_	2.0516	↑
6	3-Hydroxy-3-methylpentane-1,5-dioic acid	Amino acid and derivatives	C_6_H_10_O_5_	1.9844	↑
7	Neocnidilide	Lactones	C_12_H_18_O_2_	1.8927	↑
8	N-Acetyl-L-glutamate 5-semialdehyde	Carboxylic acids	C_7_H_11_NO_4_	1.8650	↑
9	L-Gulose	Carbohydrates	C_6_H_12_O_6_	1.8193	↑
10	Naringenin	Flavonoids	C_15_H_12_O_5_	1.6946	↑
11	N6-isopentenyladenosine	Phytohormone	C_15_H_21_N_5_O_4_	1.4313	↑
12	Quercetin 3-O-neohesperidoside	Flavonoids	C_27_H_30_O_16_	2.2128	↓
13	Rutin	Flavonoids	C_27_H_30_O_16_	2.2006	↓
14	Saccharate	Organooxygen compounds	C_6_H_10_O_8_	2.1704	↓
15	6-Gingerol	Phenols	C_17_H_26_O_4_	2.1281	↓
16	Cleomiscosin A	Coumarins	C_20_H_18_O_8_	2.1241	↓
17	Gentisic acid	Phenols	C_7_H_6_O_4_	2.1208	↓
18	Isoquercitrin	Flavonoids	C_21_H_20_O_12_	2.0966	↓
19	Chlorogenic acid	Phenylpropanoids	C_16_H_18_O_9_	2.0955	↓
20	Cynarin; 1,5-Dicaffeoylquinic acid	Organic acids	C_25_H_24_O_12_	2.0374	↓
21	Tricetin	Flavonoids	C_15_H_10_O_7_	2.0285	↓
22	Kaempferol-3-O-rutinoside	Flavonoids	C_27_H_30_O_15_	1.9922	↓
23	Esculin	Coumarins	C_15_H_16_O_9_	1.9670	↓
24	Kaurenoic acid	Diterpenes	C_20_H_30_O_2_	1.8277	↓
25	Ponasterone A	Steroids	C_27_H_44_O_6_	1.7986	↓
26	Hyperoside	Flavonoids	C_21_H_20_O_12_	1.7832	↓
27	7-Ethyl-10-Hydroxycamptothecin	Alkaloids	C_22_H_20_N_2_O_5_	1.7391	↓
28	Homoeriodictyol	Flavonoids	C_16_H_14_O_6_	1.5835	↓
29	3-Ethoxy-4-hydroxybenzaldehyde	Phenols	C_9_H_10_O_3_	1.5441	↓
30	2′,3,5,7-Tetrahydroxyflavone	Flavonoids	C_15_H_10_O_6_	1.4038	↓

Note: “↑” indicates that the content in CMF is higher than that in CMD; “↓” indicates that the content in CMF is lower than that in CMD.

**Table 2:** Top ten core targets.

Gene Name	Degree	Ingredient Name	Category
*TNF*	310	Quercetin 3-O-neohesperidoside	Flavonoids
		Rutin	Flavonoids
		Isoquercitrin	Flavonoids
		Kaempferol-3-O-rutinoside	Flavonoids
		Hyperoside	Flavonoids
		Esculin	Coumarins
*IL6*	294	Ponasterone A	Steroids
		6-Gingerol	Phenols
*ALB*	272	Gentisic acid	Phenols
*AKT1*	270	Tricetin	Flavonoids
		Quercetin	Flavonoids
		3-Hydroxycoumarin	Coumarins
		2′,3,5,7-Tetrahydroxyflavone	Flavonoids
*IL1B*	258	Neocnidilide	Lactones
*IL-10*	45	Naringenin	Flavonoids
*SRC*	226	Tricetin	Flavonoids
		3-Hydroxycoumarin	Coumarins
		Homoeriodictyol	Flavonoids
*TP53*	224	Kaurenoic acid	Diterpenes
*EGFR*	212	Tricetin	Flavonoids
*CTNNB1*	206	(+)-Abscisic acid	Phytohormone

## Data Availability

Data are contained within the article and Supplementary Materials.

## Supplementary Materials

The following supporting information are available online: https://www.mdpi.com/article/10.3390/ijms26178678/s1.
