# Supplementary material for: Neocnidilide and 6-Gingerol as Key Bioactives in Fresh and Dried Centipeda minima: Distinct Th1/Th2 Modulation via NF-κB/JAK-STAT Pathways for Allergic Rhinitis Therapy
Source: Int J Mol Sci. 2025 Sep 5;26(17):8678. doi: 10.3390/ijms26178678 (PMC12429449; doi:10.3390/ijms26178678)
Supplement: Supplementary file 1 [file ijms-26-08678-s001.zip › ijms-3696953-supplementary.pdf]

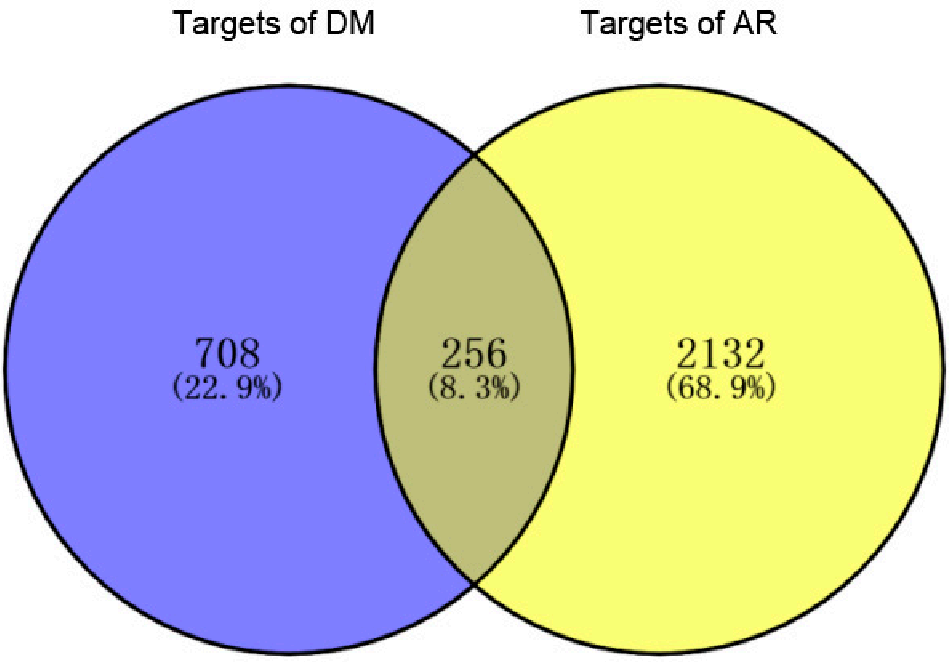

Figure S1. Venn diagram of differential metabolites-AR-target interactions. Blue circles represent the number of targets corresponding to differential metabolites (DM) in CMF vs. CMD, while yellow circles represent the number of targets associated with allergic rhinitis (AR).

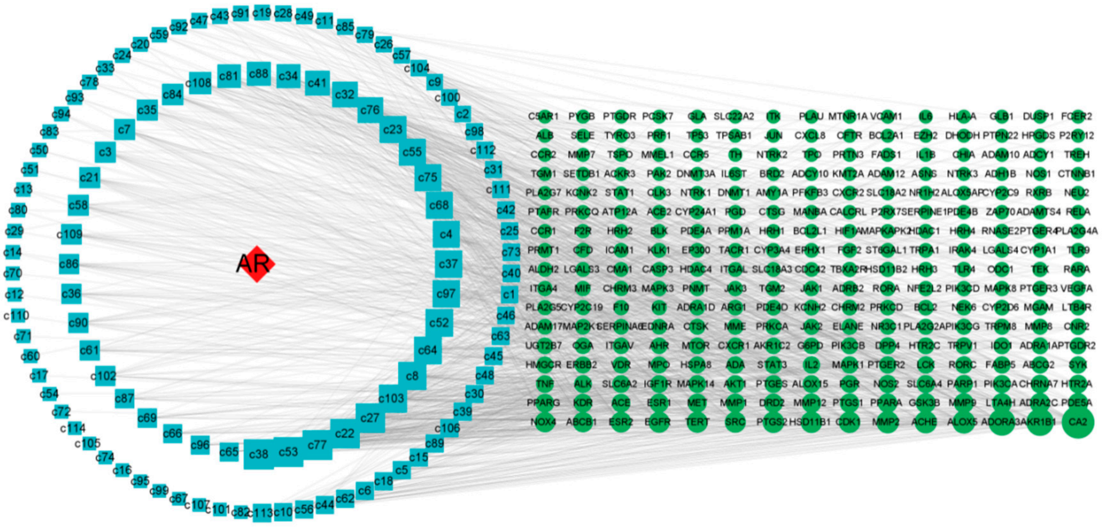

Figure S2. Compound-disease-target interaction network. Red diamonds represent allergic rhinitis (AR), blue rectangles represent differential metabolites, and green ellipses represent corresponding targets.

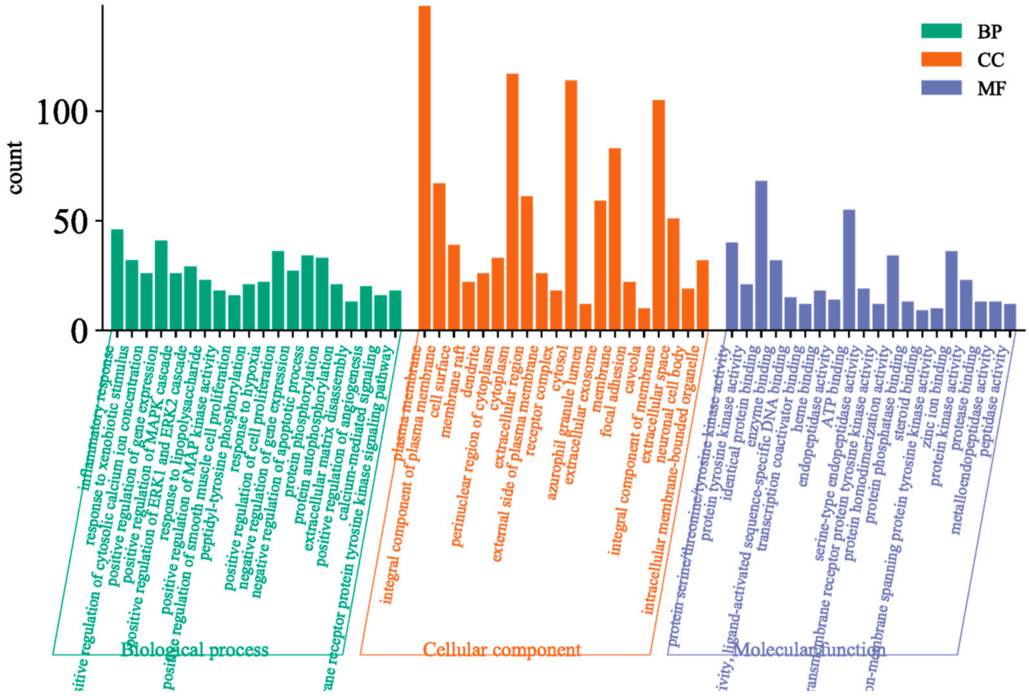

Figure S3. Gene Ontology (GO) enrichment analysis of network pharmacology. BP: Biological Process, CC: Cellular Component, MF: Molecular Function.

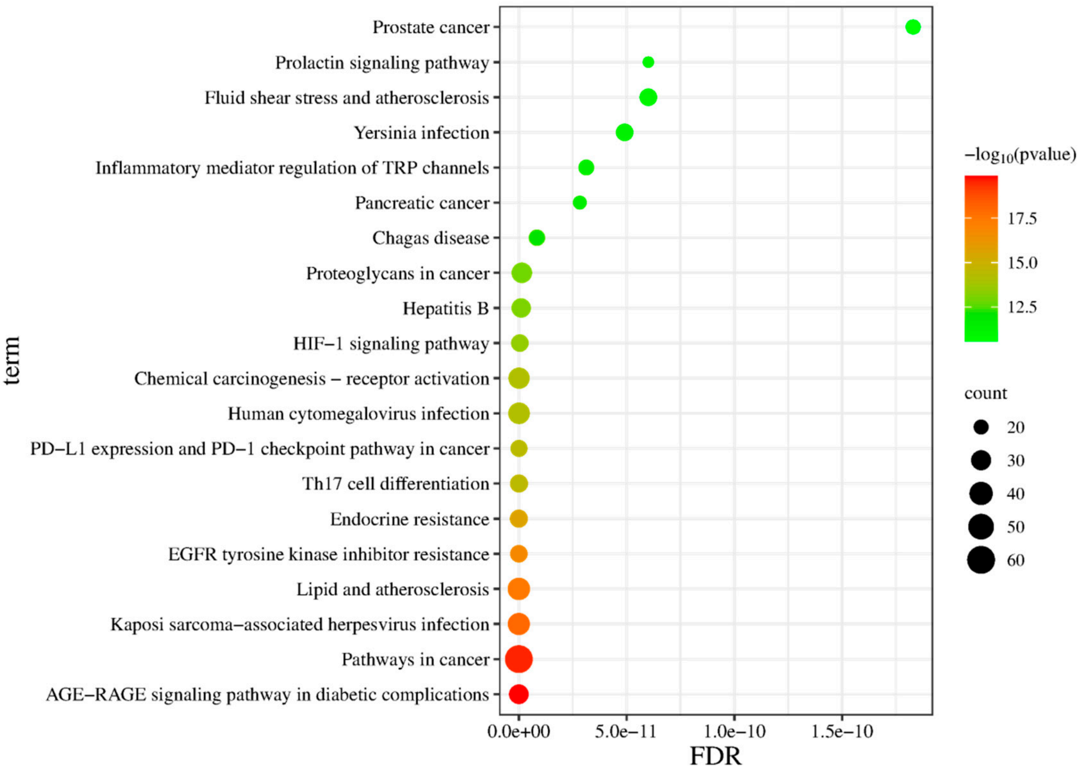

Figure S4. KEGG pathway enrichment analysis of network pharmacology. Abscissa (X-axis) represents the false discovery rate (FDR) value of each pathway; Ordinate (Y-axis) displays the KEGG metabolic pathways.The size of the dots corresponds to the number of differentially enriched metabolites within the pathway. Color intensity reflects the magnitude of −log10P-value, where deeper red indicates a higher −log10 P-value and denotes more statistically significant enrichment.
